# Leptin Receptor Overlapping Transcript (LEPROT) Is Associated with the Tumor Microenvironment and a Prognostic Predictor in Pan-Cancer

**DOI:** 10.3389/fgene.2021.749435

**Published:** 2021-11-11

**Authors:** Bingsheng Li, Yao He, Pan Li, Xiang Chen

**Affiliations:** ^1^ Department of Urology, Xiangya Hospital, Central South University, Changsha, China; ^2^ Department of Urology, University Hospital Munich, LMU Munich, Munich, Germany; ^3^ Institute for Pathology of the Ludwig-Maximilians-Universität München, Munich, Germany

**Keywords:** leptin receptor overlapping transcript, tumor microenvionment, tumor immune cell infiltration, inflammation pathways, immunotherapy, bioinformactics

## Abstract

**Background:**

Leptin receptor overlapping transcript (LEPROT) is reported to be involved in metabolism regulation and energy balance as well as molecular signaling of breast cancer and osteosarcoma. LEPROT is expressed in various tissue and is suggested to be involved in cancer developments but with contradictory roles. The comprehensive knowledge of the effects of LEPROT on cancer development and progression across pan-cancer is still missing.

**Methods:**

The expressions of LEPROT in cancers were compared with corresponding normal tissues across pan-cancer types. The relationships between expression and methylation of LEPROT were then demonstrated. The correlations of LEPROT with the tumor microenvironment (TME), including immune checkpoints, tumor immune cells infiltration (TII), and cancer-associated fibroblasts (CAFs), were also investigated. Co-expression analyses and functional enrichments were conducted to suggest the most relevant genes and the mechanisms of the effects in cancers for LEPROT. Finally, the correlations of LEPROT with patient survival and immunotherapy response were explored.

**Results:**

LEPROT expression was found to be significantly aberrant in 15/19 (78.9%) cancers compared with corresponding normal tissues; LEPROT was downregulated in 12 cancers and upregulated in three cancers. LEPROT expressions were overall negatively correlated with its methylation alterations. Moreover, LEPROT was profoundly correlated with the TME, including immune checkpoints, TIIs, and CAFs. According to co-expression analyses and functional enrichments, the interactions of LEPROT with the TME may be mediated by the interleukin six signal transducer/the Janus kinase/signal transducers and activators of the transcription signaling pathway. Prognostic values may exist for LEPROT to predict patient survival and immunotherapy response in a context-dependent way.

**Conclusions:**

LEPROT affects cancer development by interfering with the TME and regulating inflammatory or immune signals. LEPROT may also serve as a potential prognostic marker or a target in cancer therapy. This is the first study to investigate the roles of LEPROT across pan-cancer.

## Introduction:

Cancer is one of the leading causes of death, and the incidence and mortality are rapidly growing worldwide (Bray et al., 2018; Siegel et al., 20192019; Sung et al., 2021). A better understanding of the complex molecular signals of cancer initiation and progression will facilitate the identification of targeted therapeutic approaches.

Cancer cells are avid fans of making more copies of themselves, but this aberrant proliferation is accompanied by an extreme environment for cells, for instance, hypoxia and an inadequate blood supply due to abnormal vascularization. Therefore, the molecular “survival of the fittest” scenario plays out in cancer cells. In order to survive, they acquire a number of characteristic alterations, such as maintaining certain genetic variants that confer advantages on clones, altering the pathways in cellular metabolism, and developing unique ways of interacting with the microenvironment (Weinberg, 2007). To achieve these changes, cancer cells hijack and reprogram the existing signaling circuitry, which is the foremost system of communication among cells, rather than demolishing the entire system. Thus, signaling pathways might generate different outcomes in normal and cancer cells or even different cell types; the activation of signaling molecules may also have different consequences, depending on the cellular environment (Weinberg, 2007). Therefore, pan-cancer studies of signaling pathways and signaling molecules could provide a better overall understanding of their roles in cancer initiation and progression and be helpful for identifying potential therapeutic targets.

Leptin signaling is key in regulating energy balance and metabolic homeostasis in normal cells (Myers et al., 2008; Stern et al., 2016). It is suggested to be also involved in cancer development by mediating the Janus kinase/signal transducers and activators of transcription (JAK/STAT) cascade pathway (Mullen and Gonzalez-Perez, 2016), which induces proliferation and angiogenesis of cancer cells (Mullen and Gonzalez-Perez, 2016; Wang et al., 2018) and regulates the immune responses to tumors (Zhang et al., 2020a). Leptin exerts its biological action majorly through binding to and activating the leptin receptors (LEPR) (Garofalo and Surmacz, 2006; Ghasemi et al., 2019), and the gene encoding LEPR overlapping transcript (LEPROT), which is shown to negatively regulate leptin signaling by reducing expressions of LEPR on the cell membrane, is considered as an anticancer factor in cancer progression. However, a more recent study paradoxically reported that LEPROT could activate the JAK/STAT signaling (Li et al., 2019), which may theoretically facilitate cancer development (Mullen and Gonzalez-Perez, 2016). Therefore, the role of LEPROT in tumors may not be limited to the previous cognition that it only serves as a negative regulator of leptin signaling.

Because of conflicting reports on the impacts of LEPROT on downstream signaling and cancer progression, and the lack of investigation on it, we aimed to illustrate the role of LEPROT in tumor initiation and tumor progression across multiple cancer types by bioinformatic analysis. In the current study, we first provide a full description of the LEPROT expression in pan-cancer and their molecular subtypes as well as the correlation between LEPROT and the tumor microenvironment (TME), in particular, the immune cells infiltration and cancer-associated fibroblasts (CAFs). In addition, to better understand the molecular mechanisms of LEPROT, the most relevant molecules and signaling pathways of LEPROT were identified.

## Materials and Methods

### Data Sources and Processing

The gene expression data of LEPROT and clinical follow-up information in pan-cancer atlas studies were extracted from The Cancer Genome Atlas (TCGA) database (Tomczak et al., 2015) using the cBio cancer genomics portal (cBioPortal) cBioPortal server (https://www.cbioportal.org/) for further analysis (Cerami et al., 2012; Gao et al., 2013). Apart from the expression profiles of TCGA, the Genotype-Tissue Expression (GTEx) database, which builds on molecular profiling of nondiseased tissue sites; the NCBI’s Gene Expression Omnibus (GEO), which stores curated gene expression data sets and might serve as a complement of TCGA data sets; and the European Genome-phenome Archive (EGA) are also included in the current study. The abbreviations of the types of cancers examined in our analyses are as follows: ACC: adrenocortical carcinoma; BLCA: bladder urothelial carcinoma; BRCA: breast invasive carcinoma; CESC: cervical squamous cell carcinoma; CHOL: cholangiocarcinoma; COAD: colon adenocarcinoma; CRC: colorectal carcinoma; DLBC: lymphoid neoplasm diffuse large B cell lymphoma; ESCA: esophageal carcinoma; GBM: glioblastoma multiforme; LGG: brain lower grade glioma; HNSC: head and neck squamous cell carcinoma; KICH: kidney chromophobe; KIRC: kidney renal clear cell carcinoma; KIRP: kidney renal papillary cell carcinoma; LAML: acute myeloid leukemia; LIHC: liver hepatocellular carcinoma; LUAD: lung adenocarcinoma; LUSC: lung squamous cell carcinoma; MESO: mesothelioma; OV: ovarian serous cystadenocarcinoma; PAAD: pancreatic adenocarcinoma; PCPG: pheochromocytoma and paraganglioma; PRAD: prostate adenocarcinoma; READ: rectum adenocarcinoma; SARC: sarcoma; SKCM: skin cutaneous melanoma; STAD: stomach adenocarcinoma; TGCT: testicular germ cell tumors; THCA: thyroid carcinoma; THYM: thymoma; UCEC: uterine corpus endometrial carcinoma; UCS: uterine carcinosarcoma; and UVM: uveal melanoma; STES: stomach and esophageal carcinoma cohort; KIPAN, Pan-kidney cohort (KICH + KIRC + KIRP). Plots in the study were conducted in Graphpad Prism 9.0, R version 4.0.5, or downloaded from online servers and then compiled and edited by Affinity Designer 1.8.5. All data or analyses are in accordance with the latest version in July 2021.

### LEPROT Expression in Tumor and Normal Tissue or Among Different Cancer Molecular Subtypes

The Human Protein Atlas (HPA, https://www.proteinatlas.org/) is a comprehensive resource for the exploration of the human proteome, and it contains information of mRNA expression and protein level in cells, tissues, and organs (Uhlén et al., 2015). The LEPROT expression level in normal tissues was achieved from HPA based on the GTEx database. An overview plot to compare LEPROT mRNA expressions in tumor tissues and corresponding normal tissues across TCGA cancer types was achieved by the “Cancer Exploration” > “Gene_DE” module of TIMER2.0 (http://timer.comp-genomics.org/) (Li et al., 2020). TIMER is currently using the expression data of tumor tissues and the corresponding normal tissues in TCGA of the latest version. Differences in LEPROT mRNA expression between the tumor and the corresponding normal tissues were also shown by boxplots generated by the UALCAN (http://ualcan.path.uab.edu/analysis.html), a web portal for analyses, including expression distributions according to data from the TCGA and Clinical Proteomic Tumor Analysis Consortium (CPTAC) data set (Chandrashekar et al., 2017; Chen et al., 2019a). Expression of LEPROT (presented as log2 [counts per million (CPM)]) among molecular subtypes across pan-cancer were checked by the “Subtype” > “Molecular subtypes” module of TISIDB portal (http://cis.hku.hk/TISIDB/index.php) (Ru et al., 2019) with all 17 available cancer types analyzed, including ACC, BRCA, COAD, ESCA, GBM, HNSC, KIRP, LGG, LIHC, LUSC, OV, PCPG, PRAD, READ, SKCM, STAD, and UCEC, and those significant ones presented (*p*-value < .05). Mutation features of LEPROT in the TCGA pan-cancer samples were analyzed using the cBioPortal. “TCGA Pan Cancer Atlas Studies” was selected before “LEPROT” was queried in “Query by gene” to get the results of the gene alteration patterns across pan-cancer types in the “Cancer Types Summary” module.

### Correlations of LEPROT Expression and Methylation

LinkedOmics (http://www.linkedomics.org/) is a web portal for comprehensive multigroup data analysis for multiple cancer types (Vasaikar et al., 2018). The relationship of LEPROT DNA methylation (methylation preprocessor, presented as beta values (value-0.5) with its mRNA expression (presented as RNA-Seq by Expectation-Maximization (RSEM), Log2 (value + 1)) across cancer types was analyzed using the LinkedOmics with Methylation450 and high-output RNA sequencing (HiSeq) data. Pearson correlation was conducted for a correlation coefficient R and a *p*-value for each cancer type.

### Gene Expression Correlation Analysis

Partial correlation analysis between LEPROT and expression or mutation status of certain genes was conducted using the “Exploration” > “Gene_Corr”/“Gene_Mutation” module of TIMER2.0. The heatmap of the correlations shows the coefficient of Spearman correlation with red indicating a statistically significant positive association and blue indicating a statistically significant negative association (significant when *p* < .05); gray denotes a nonsignificant result. The scatterplots were based on log2 [TPM]. The cBioportal was used to identify the top 500 LEPROT-correlated genes and to generate scatterplots of the correlations of LEPROT and genes of interest across cancer types, via selecting each “PanCancer Atlas” study before “LEPROT” was queried in “Query by gene” to obtain the top correlated genes with LEPROT in the “coexpression” module. The intersection of the top 500 correlated genes among different cancer types was obtained and visualized using the Upset tool by R package “UpSetR” (Lex et al., 2014).

### The TME Estimation

The stromal and immune scores were obtained by the Estimate algorithm (https://bioinformatics.mdanderson.org/estimate/) (Yoshihara et al., 2013). A higher stromal or immune score indicates a larger number of stromal or immune components; the ESTIMATE score is the sum of the stromal and immune scores. The relationship between stromal/immune/ESTIMATE scores and LEPROT expression is indicated by the Pearson correlation coefficient and visualized by R package “ggplot2” (Wickham, 2016). The relationship between LEPROT and infiltrating CD4^+^, CD8^+^, regulatory T cells, and CAFs are demonstrated by the “Immune” > “Gene” module of TIMER2.0 with results conducted by different algorithms, including TIMER (Li et al., 2016), xCell (Aran et al., 2017), MCP-counter (Becht et al., 2016), CIBERSORT (Newman et al., 2015), EPIC (Racle et al., 2017), and quanTIseq (Finotello et al., 2019). Overall correlations of LEPROT expression and gene signatures (including signatures of specific immune cells or CAFs) using expression data from all cancer samples were generated by the “Correlation Analysis” module of the web server GEPIA2 (http://gepia2.cancer-pku.cn/) (Tang et al., 2019).

### Protein–Protein Interaction and Enrichment Analysis

Gene Set Enrichment Analysis (GSEA) software (Subramanian et al., 2005) was established to determine the statistically significant gene sets in the comparison between different subgroups. GSEA (Version 4.10) was obtained from the Broad Institute (http://software.broadinstitute.org/gsea/index.jsp). Expression profiles of cancer studies were pre-ranked according to the Pearson correlation coefficient with LEPROT and input with the annotation file “H: hallmark gene sets” and “C5: gene ontology sets”. The cutoff values were predefined as FDR <0.25. GeneMANIA (http://www.genemania.org) (Warde-Farley et al., 2010) was used to construct protein–protein interaction networks of certain proteins and related proteins and enrich the co-regulated genes into functions. The proteins were connected based on “physical interactions,” “co-expression,” “predicted co-localization,” “genetic interactions,” and “pathway shared” protein domains.”

### Survival Analysis

Survival analysis was conducted using R package “ggplot2” (Wickham, 2016) to get forest plots showing the hazard ratio and *p*-value for overall survival (OS) or disease-specific survival (DSS) in patients across cancer types in TCGA, based on LEPROT high versus low expression, in the Sangerbox tools, a free online platform for data analysis (http://www.sangerbox.com/tool). Kaplan–Meier analysis of OS or recurrence-free survival (RFS) was obtained using the Kaplan–Meier plotter (KM-plotter) (www.kmplot.com) (Nagy et al., 2021), using “mRNA” > “Start KM Plotter for pan-cancer” module with the setup of “split patients by auto select best cutoff” to divide LEPROT mRNA into high and low groups.

### Correlations of LEPROT Expression and Patient Responses to Immune Checkpoints Inhibitors Therapy

Gene expression profile and corresponding clinical responses to immune checkpoint inhibitors (CPIs) in GSE67501 and GSE79691 data sets were downloaded from the GEO database (https://www.ncbi.nlm.nih.gov/gds). The associations of LEPROT expression and patient responses to CPIs were investigated by comparing LEPROT-low (lower than the median) and LEPROT-high (higher than the median) groups using the Mann–Whitney U test. A simple logistic regression was also conducted to generate a receiver operating curve (ROC). Graphpad Prism 9.0 was used to conduct statistics.

## Results

### LEPROT is Aberrantly Expressed in Human Cancers

As LEPROT was expressed widely in human tissues (Uhlén et al., 2015), we first explored the level of LEPROT in different tissues, especially in normal and tumor tissues across organs. By exploring the HPA and GTEx databases, LEPROT was found to be highly expressed in female genital organs, including the endometrium, cervix, and placenta and also in the kidney (Supplementary Figure S1A). The mRNA level of LEPROT was various in different tissues with relatively low tissue and low cell-type specificity (Supplementary Figure S1A).

**FIGURE 1:**
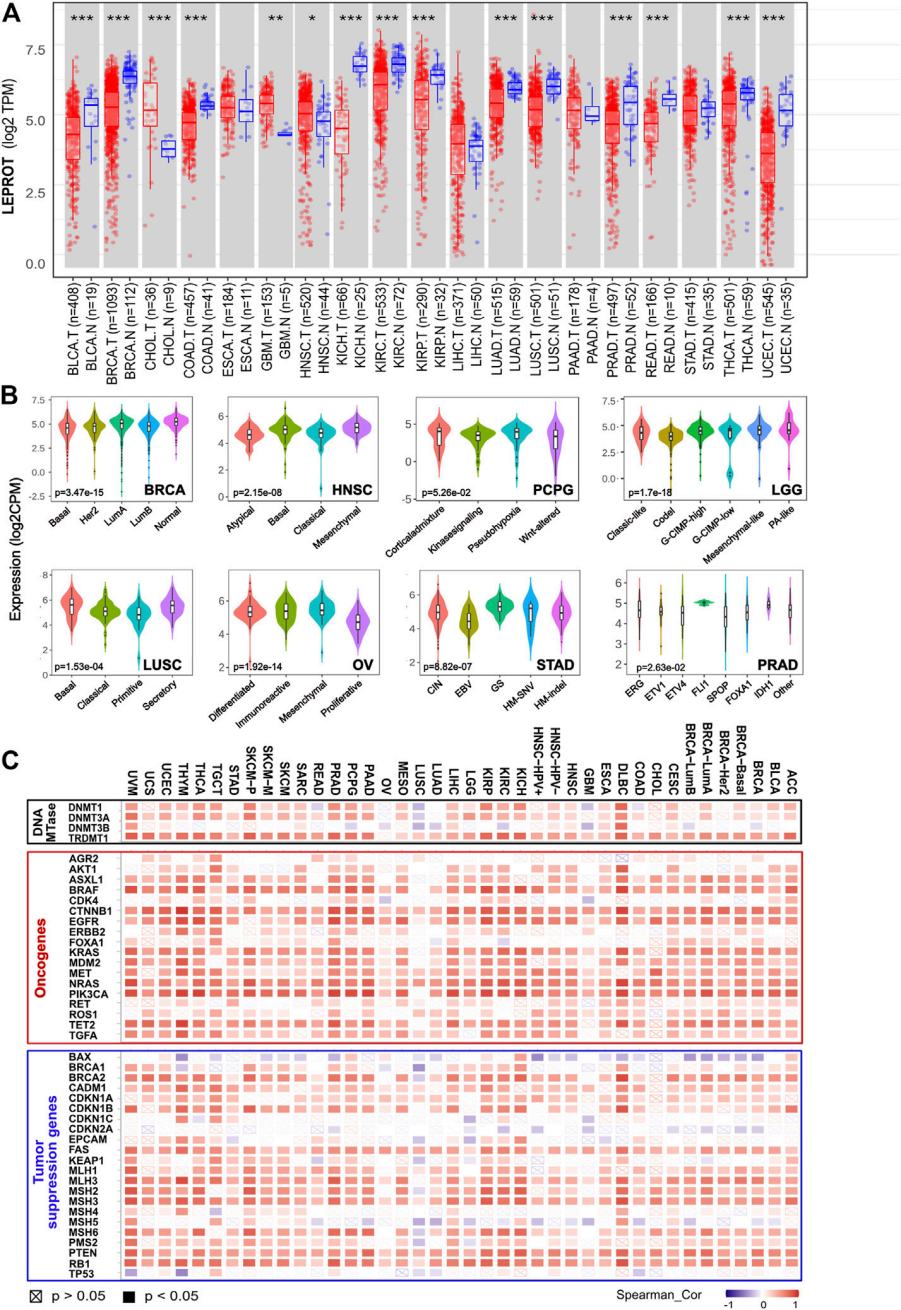
Aberrant expression of LEPROT gene in different tumors and its correlation with methylation enzyme genes and mismatch repair genes. **(A)** The expression status of the LEPROT gene in different cancers or specific cancer subtypes was compared with corresponding normal tissues through TIMER2. *p < .05; **p < .01; ***p < .001. **(B)** The violin plots of the LEPROT gene expression in different molecular subtypes of LGG, BRCA, OVA, STAD, LUSC, and PRAD (*p* < .05 among the subtypes). **(C)** Pearson correlation analysis of LEPROT with expressions of DNA methyltransferases (upper panel), oncogenes (middle panel), and tumor-suppressor genes (lower panel) in pan-cancer types.

Moreover, a comparison between tumor tissues and adjacent normal tissues showed the LEPROT mRNA levels were significantly lower in the tumor tissues of BLCA, BRCA, COAD, KICH, KIRC, KIRP, LUAD, LUSC, PRAD, READ, THCA, and UCEC (*p* < .001) and higher in CHOL (*p* < .001), GBM (*p* < .01), and HNSC (*p* < .05) (Figure 1A) compared with the corresponding normal tissues. Thirteen cancers (ACC, DLBC, LAML, LGG, MESO, OV, PCPG, SARC, SKCM, TGCT, THYM, UCS, UVM, UCEC, and PCPG) were removed from the comparison analysis due to a lack of corresponding normal tissue or a normal tissue sample of less than three cases. Similar results were also observed in the UALCAN (Supplementary Figure S1B). Further evaluation of LEPROT mRNA levels was performed in different molecular subtypes across pan-cancer types. LEPROT was found to be distinctly expressed in different molecular subtypes of LGG, BRCA, OV, HNSC, STAD, LUSC, PRAD, and PCPG (*p* < .05) (Figure 1B). Specifically, LEPROT was expressed in a significantly lower level in the proliferative subtype of OV (*p* < .05) and in a significantly higher level in the mesenchymal subtype of HNSC (*p* < .05) (Figure 1B), suggesting that LEPROT might be related to more aggressive subtypes.

To investigate whether the alterations of expression or functional roles of LEPROT resulted from its mutations, the mutation feature of LEPROT in TCGA pan-cancer atlas was examined and demonstrated in Supplementary Figure S2A. The pan-cancer mutation spectra of LEPROT indicated a low mutant frequency of LEPROT across all cancer types with the maximal alteration frequency of LEPROT in SARC (only 3.14%). There was no significant difference between the expression of LEPROT in wild-type and mutated cases except for the limited LEPROT-mutated cases (n = 2) in COAD that had a lower level of LEPROT compared with LEPROT wild-type cases (*p* < .05) (Supplementary Figure S2B). These results indicate LEPROT was aberrantly expressed in cancer tissues, but it did not result from its mutations in most cancer types.

### Correlations of LEPROT Expression With its Methylation Level and DNA Methylation Transferases Expression Across TCGA Pan-Cancer Types

To interpret the cause of low LEPROT expression across cancer types, DNA methylation of LEPROT, the epigenetic modifications that altered gene expression, were investigated. First, correlations of LEPROT expression and methylation level were examined by LinkedOmics. It revealed LEPROT methylation reduced its expression in most cancer types except for ACC, BLCA, HNSC, KICH, MESO, SKCM, and UCEC (Supplementary Figure S3). The negative correlations between the expression of LEPROT and its methylation were most significant in OV (Pearson R = -0.745, *p* = .02) and CHOL (Pearson R = -0.523, *p* = .001). In addition, as key factors in regulating DNA methylation levels (Cui and Xu, 2018), the correlation of the DNA methyltransferase (DNMT) family (DNMT1, DNMT2, DNMT3A, DNMT3B) and LEPROT expression were also investigated. Unexpectedly, the expression of LEPROT was positively correlated with that of DNMT1 and DNMT3A but not DNMT3B in most cancers (Figure 1C) and was consistently positive-correlated with DNMT2 in all cancers. Therefore, we further examined the correlation between DNMTs and methylation level of LEPROT, and the results show no significant correlations, represented by DLBC and OV (Supplementary Figure S3B, C). These results suggest that the aberrant expression of LEPROT might be regulated by DNA methylations, but the methylation level of LEPROT was regulated independent of DNMTs.

### LEPROT is Correlated With Tumor Suppressor Genes Across TCGA Pan-Cancer Types

Tumorigenesis is often driven by mutation or compromising of tumor-suppressor genes, which restricted the cell cycle and repaired DNA (Lee and Muller, 2010; van der Weyden et al., 2012). Although, generally, mutations of tumor suppressor genes did not result in the alteration of LEPROT expression (Supplementary Figure S4A), the expression of tumor-suppressor genes is shown to be highly related to LEPROT expression. Tumor-suppressor genes BAX, CDKN2A, and MSH5 were negatively correlated with LEPROT in multiple cancer types, and most tumor-suppressor genes were suggested to be positively correlated with LEPROT in a gene-consistent way across cancer types (Figure 1C). Notably, as tumor-suppressor genes, mismatch repair (MMR) genes constitute the MMR system that recognizes and repairs DNA mistakes (Marti et al., 2002; Putnam, 2016), six of which (MLH1, MLH3, MSH2, MSH3, MSH6, and PMS2) was overall positively correlated with LEPROT in mRNA expression. These results suggest that the aberrant expression LEPROT may be accompanied by abnormal tumor suppressor gene functions, especially compromised DNA mismatch repair.

### LEPROT is Positively Correlated With Oncogenes

Oncogenes are genes that are abnormally expressed and have the potential to drive cancer. To understand the role of the aberrantly expressed LEPROT in cancer development, we explored the correlation between the mRNA expression level of LEPROT and commonly reported oncogenes across various cancer types by TIMER2. As shown in Figure 1C, LEPROT expression was positively correlated with most oncogenes in a broad spectrum of cancer types, suggesting a role of LEPROT in promoting cancer progression. However, mutations of oncogenes did not result in the alteration of LEPROT expression (Supplementary Figure S4B), which indicated that mutations of those oncogenes did not drive expression alterations of LEPROT.

### Correlations of LEPROT With TME

**FIGURE 2:**
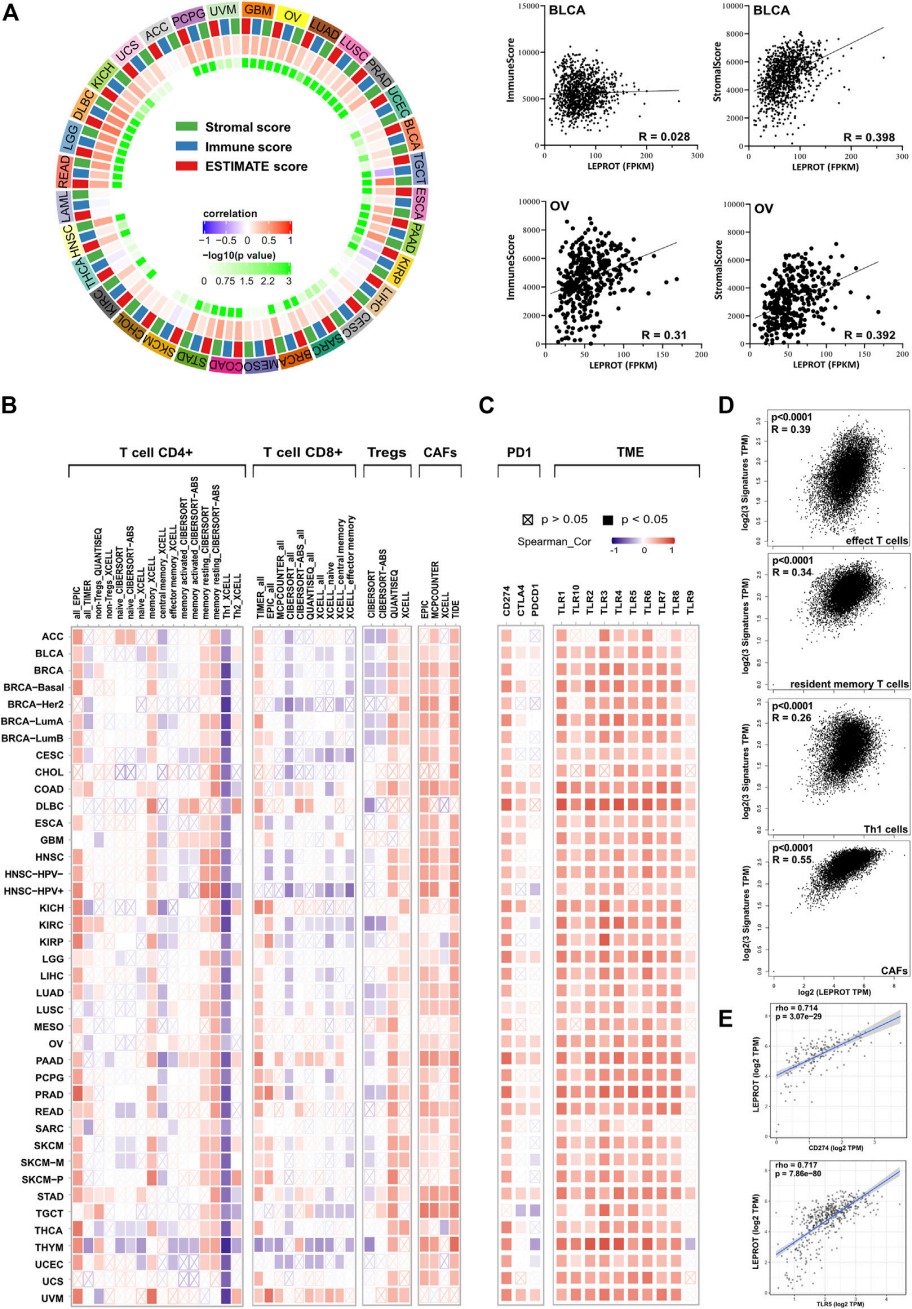
Correlation analysis between LEPROT expression and TME conditions. **(A)** Correlations between LEPROT expression and stromal, immune, and ESTIMATE scores were conducted using Pearson correlation for each cancer type; the innermost cycle represents *p*-values of the Pearson correlation, and the middle cycle represents correlation coefficient. Some representative scatterplotsare demonstrated in the right panel. **(B)** Correlation heatmaps using different algorithms showing the potential correlation between the expression level of the LEPROT gene and the infiltration level of CD4^+^, CD8^+^, and regulatory T cells as well as CAFs across all types of cancer in TCGA, analyzed by TIMER2. **(C)** Correlation heatmaps showing the correlation between the expression level of the LEPROT gene and TME genes, including PD-L1, CTLA-4, PD-1, and TLR1-10 across all types of cancer in TCGA analyzed by TIMER2. **(D)** Correlation plots showing the correlation between the expression level of the LEPROT gene and signatures of different infiltrated immune cells across all types of cancer in TCGA, analyzed by GEPIA2. **(E)** Scatter correlation plots showing the correlation between LEPROT and PD-L1 in PAAD (upper panel) and between LEPROT and TLR5 in PRAD (lower panel) as representatives for the heatmap in (C).

As a member of the leptin signaling pathway, LEPROT was not only involved in the regulation of intracellular signaling pathways but, more importantly, was involved in the interaction of tumor cells with the extracellular TME, which played a regulatory role in tumor progressing through reversible changes in signal transduction or gene expression programs. Thus, we next estimated the association of LEPROT and TME components. Stromal score, which was calculated based on gene signature, was a direct quantitative indicator of stromal cell infiltration in the microenvironment. Thus, the relationship between LEPROT expression and stromal scores was first estimated. Pearson correlation analysis showed LEPROT expression was positively correlated with the stromal score in 23/33 cancers (69.7%; they were BLCA, BRCA, CESC, COAD, DLBC, ESCA, GBM, HNSC, KICH, KIRC, LGG, LIHC, LUAD, LUSC, OV, PAAD, PCPG, PRAD, READ, SARC, STAD, TGCT, and UCEC (*p* < .05)) without any significant negative correlation among TCGA cancer types (Figure 2A).

To better understand which stromal components were positively associated with LEPROT, we checked the relationship between LEPROT mRNA level and immune cell infiltration (TII). As the most important compositions of the stromal, the ratio of TII reflected by immune score was calculated. It was positively correlated with LEPROT expression in COAD, DLBC, GBM, HNSC, KICH, LGG, LIHC, LUAD, LUSC, OV, PAAD, PCPG, PRAD, READ, STAD, and UVM and negatively associated with LEPROT only in TGCT and KIRP (Figure 2A). These results aroused our interest because the composition of TII cells could serve as biomarkers for predicting patient responses to treatment and survival in terms of chemotherapy and immunotherapy (Zhang et al., 2019). As the immune score reflecting TII as a whole, the composition of TII cells was further examined, especially the distinct T cells in cancers. TIMER2, which offers many existing algorithms (Li et al., 2020; Aran et al., 2017; Newman et al., 2015; Racle et al., 2017; Li et al., 2017) for estimating tumor-infiltrating immune cell populations, showed a remarkable correlation between LEPROT expression and CD4^+^, CD8^+^, and regulatory T cells (Figure 2B). Generally, the correlation was presented in a type-dependent manner and algorithm-dependent way. But the positive relationship of LEPROT and memory CD4^+^ T cells resting, and the negative relationship of LEPROT and Th1 seemed to be strong and consistent across most cancer types. Moreover, CAFs, a prominent component of the microenvironment in most types of solid tumors, which are also considered to be involved in TII (Barrett and Puré, 2020), were also shown to consistently positively correlate with LEPROT expression regardless of the cancer types (Figure 2B). The LEPROT mRNA level was overall positively correlated with the signatures of effect T cells, resident memory T cells, Th1 cells, and CAFs across all cancer types using whole TCGA cancer data sets (Figure 2D).

Considering that LEPROT was highly associated with TII, we next investigated its association with expressions of immune checkpoint molecules (ICMs). ICMs and the composition of the TME are shown to influence each other intensively and both influence the effect of immune therapy (Jiang et al., 2019; Wang et al., 2019a; Wang et al., 2019b; Zhang et al., 2020b; Lee et al., 2019). ICMs, including programmed death 1 (PD-1), programmed cell death ligand 1 (PD-L1), and cytotoxic T lymphocyte-associated antigen 4 (CTLA-4), which played key roles in immune checkpoint inhibition, were included in the correlation analysis. As shown in Figure 2C, PD-L1 was positively correlated with LEPROT in all cancer studies, and PD-1 and CTLA-4 were limited related to LEPROT (Figure 2C). Apart from ICMs, cumulative evidence suggests profound and complex roles of toll-like receptor (TLR) signals in TME (Angrini et al., 2020). And TLR1-10 showed an overall positive correlation with LEPROT expression (Figures 2C,E). All results above suggest that LEPROT was involved in the interactions of tumor cells with TME, especially with TII and CAFs.

### Analysis of LEPROT Functional Enrichment and Interaction Network

To gain mechanistic insight into LEPROT, GSEA was performed on five TCGA studies (BLCA, BRCA, COAD, KIRC, and STAD). Pre-ranked lists of genes according to the Pearson correlation coefficient with LEPROT were adjusted for the investigation of enriched genes involving hallmarks, gene ontology (GO), and pathways. Not surprisingly, inflammatory/immune response markers and extracellular component-related pathways were enriched in these cancer types. Items significantly enriched in hallmarks were inflammatory or immune response, including IL6_JAK_STAT3 signaling, inflammatory response, complement, TNF-alpha signaling via NFKB, and IL2_STAT5 (Table 1). Items significantly enriched in GO analysis were extracellular matrix structural constituent, collagen binding of molecular functions (MF), cell matrix adhesion, cell substrate adhesion, cell substrate junction organization, and regulation of cell substrate junction organization in biological process (BP) (Table 2). These enrichment items once again illustrated the interactions of LEPROT with the TME, especially with the extracellular matrix and immune response.

**TABLE 1:** Inflammation or immune response enriched in hallmark by GSEA.

Geneset	TCGA studies	Nes	*p*-value
IL6 JAK STAT3 signaling	BLCA	2.13	0.000
	BRCA	1.80	0.000
	COAD	2.12	0.000
	KIRC	1.23	0.104^#^
	STAD	2.08	0.000
Inflammatory response	BLCA	1.88	0.000
	BRCA	2.14	0.000
	COAD	2.37	0.000
	KIRC	1.39	0.004
	STAD	2.34	0.000
Complement	BLCA	1.99	0.000
	BRCA	1.68	0.000
	COAD	1.99	0.000
	KIRC	1.31	0.017
	STAD	1.79	0.000
TNF-a siganaling via NFKB	BLCA	2.17	0.000
	BRCA	1.83	0.000
	COAD	1.93	0.000
	KIRC	0.73	1.000^#^
	STAD	2.13	0.000
IL2 STAT5 signaling	BLCA	1.48	0.000
	BRCA	1.45	0.003
	COAD	1.76	0.000
	KIRC	1.12	0.162^#^
	STAD	1.72	0.000

**TABLE 2:** Extracellular matrix enriched in GO analysis by GSEA.

Geneset	TCGA studies	Nes	*p*-value
GOMF: EXTRACELLULAR MATRIX STRUCTURAL CONSTITUENT	BLCA	2.66	0.000
	BRCA	2.49	0.000
	COAD	2.22	0.000
	KIRC	0.41	1.000^#^
	STAD	3.10	0.000
GOBP: CELL MATRIX ADHESION	BLCA	2.61	0.000
	BRCA	0.51	0.000
	COAD	1.81	0.000
	KIRC	1.93	0.000
	STAD	2.73	0.000
GOMF: COLLAGEN BINDING	BLCA	2.60	0.000
	BRCA	2.29	0.000
	COAD	2.12	0.000
	KIRC	1.15	0.188^#^
	STAD	2.57	0.000
GOBP: CELL SUBSTRATE ADHESION	BLCA	2.59	0.000
	BRCA	2.25	0.000
	COAD	1.78	0.000
	KIRC	1.79	0.000
	STAD	2.73	0.000
GOBP: CELL SUBSTRATE JUNCTION ORGANIZATION	BLCA	2.52	0.000
	BRCA	2.45	0.000
	COAD	1.66	0.000
	KIRC	2.00	0.000
	STAD	2.60	0.000
GOBP: REGULATION OF CELL SUBSTRATE JUNCTION ORGANIZATION	BLCA	2.48	0.000
	BRCA	2.50	0.000
	COAD	1.85	0.000
	KIRC	2.15	0.000
	STAD	2.46	0.000

### Analysis of LEPROT-Related Genes

**FIGURE 3:**
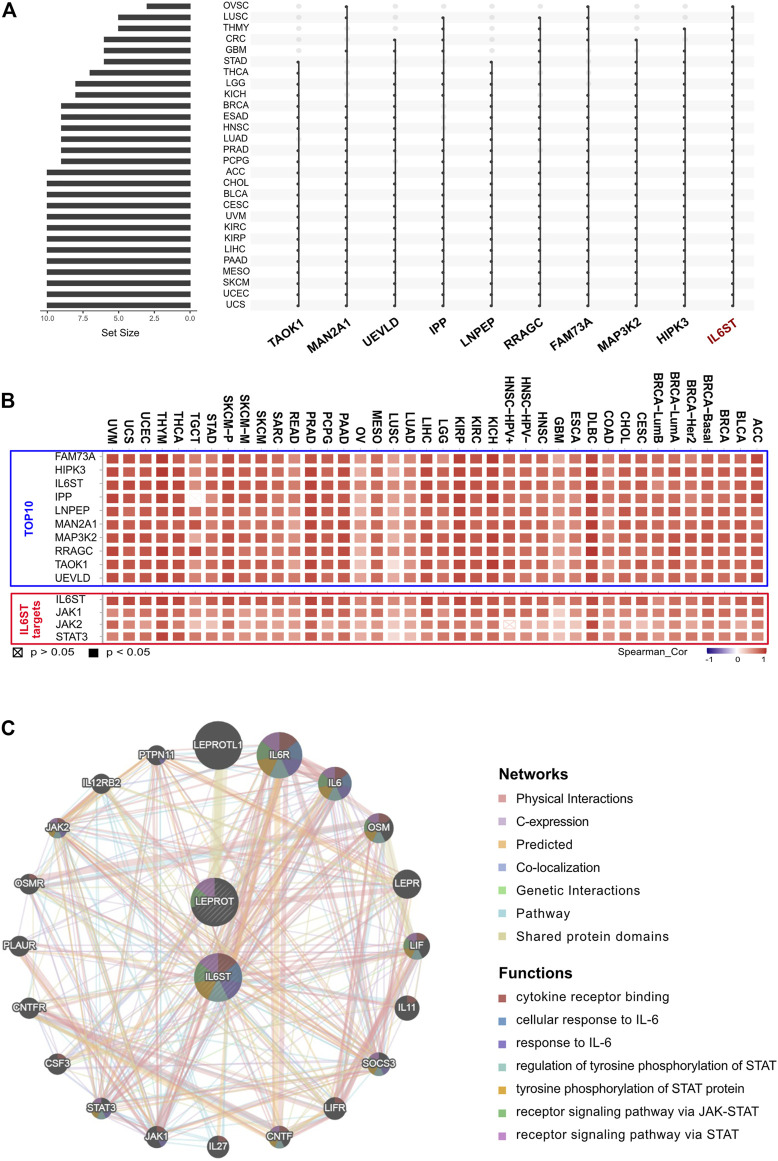
LEPROT-related gene enrichment analysis. **(A)** The interaction of top-500 related genes of LEPROT in each of 28 non-germ-cell solid cancers is shown in the upset plot, showing that genes with top-10 appearing times were IL6ST, HIPK3, MAP3K2, FAM73A, RRAGC, LNPEP, IPP, UEVLD, MAN2A1, and TAOK1. **(B)** Correlation heatmaps showing the correlation between the expression level of the LEPROT gene and those 10 genes (upper panel), and between the expression level of the LEPROT gene and IL6ST, JAK1/2, and STAT3 (lower panel). **(C)** Protein–protein interaction plot of LEPROT and IL6ST, along with their functional correlated proteins, with the enriched functions demonstrated in different colors.

To further identify the key genes that interacted with LEPROT in tumorigenesis and tumor progression, LEPROT co-regulated genes were examined using the cBioportal. Intersection analyses were performed among 28 non-germ cell solid cancers with the top 500 LEPROT-related genes (Supplementary Table S1, Figure 3A), from which IL6ST turned out to be the only intersected gene. Pearson correlation analysis was performed on the level of LEPROT and the top 10 intersected genes. The genes JAK1, JAK2, and STAT3 were also included in the analysis as they were the downstream molecules of IL6ST (Sanz-Moreno et al., 2011; Looyenga et al., 2012; Pilati and Zucman-Rossi, 2015) (Figure 3B). Results show that the level of LEPROT was consistently positively correlated with that of JAK1, JAK2, and STAT3 (Figure 3B), which suggested that JAK1/2/STAT3 might be the downstream LEPROT and mediate cancer cell proliferation and TME alterations. Moreover, to better understand the way they interacted with each other, GeneMANIA was used to construct protein–protein interaction networks of proteins encoded by LEPROT, genes highly co-regulated with IL6ST and their correlated proteins (Figure 3C). Functional enrichment analysis for these proteins suggest they were enriched in functions involving “functions cytokine receptor binding,” “cellular response to interleukin-6,” “tyrosine phosphorylation of STAT protein,” “receptor signaling pathway via JAK-STAT,” and “receptor signaling pathway via STAT.” These correlation analyses suggest that the interactions of LEPROT with the TME might be mediated by inflammatory signaling pathway IL6ST/JAK1/2/STAT3 (Figure 3C).

### Prognostic Values of the LEPROT Expression Across Cancer-Types

**FIGURE 4:**
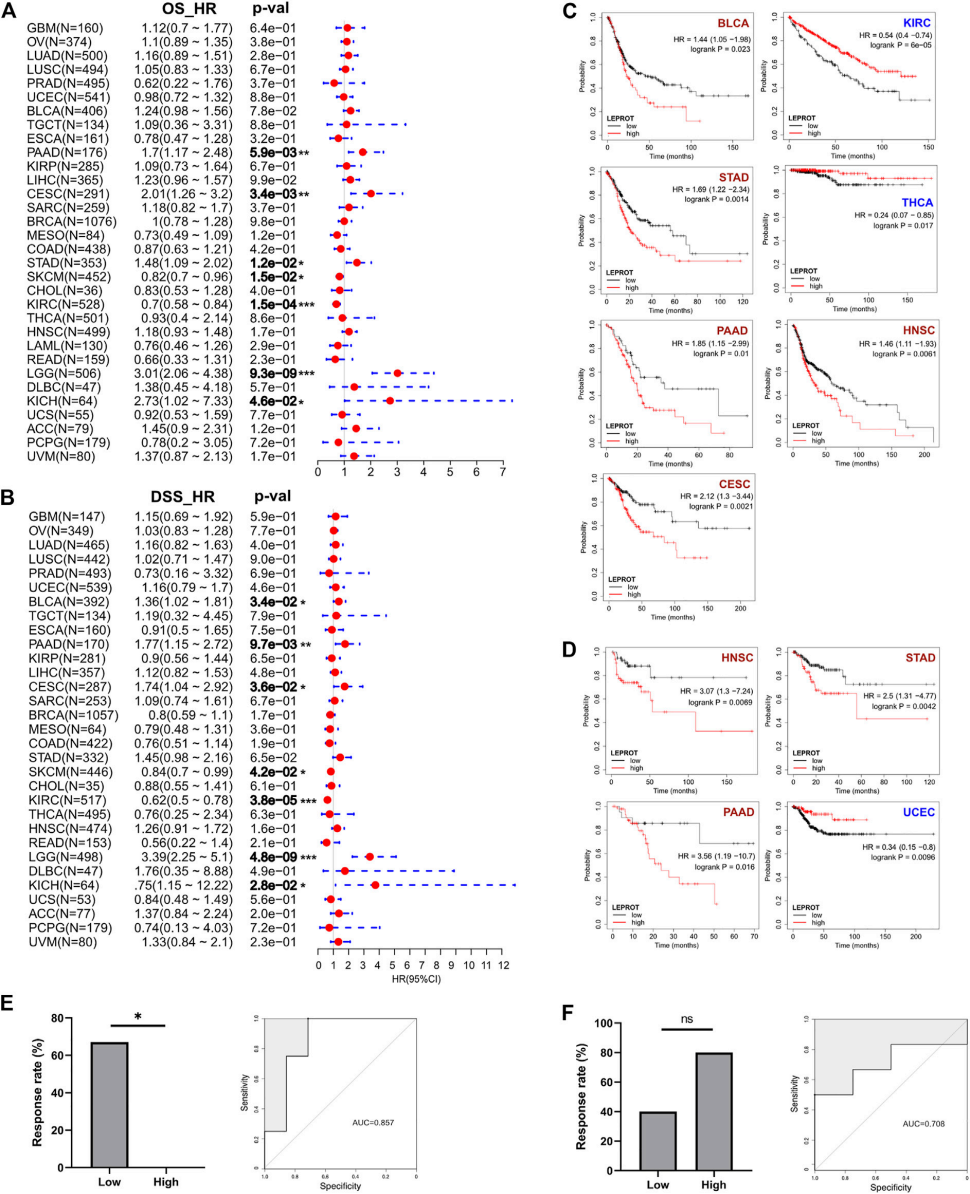
Correlation between LEPROT gene expression and survival of patients with different cancer types by best cutoff point. **(A)** Cox analyses for OS and DSS of LEPROT across pan-cancer types (left and right panels, respectively). **(B)** Kaplan–Meier curves of LEPROT gene for OS analysis with significance according to KM-Plotter. **(C)** Kaplan–Meier curves of LEPROT gene for progression-free survival analysis with significance according to KM-Plotter. **(D)** Kaplan–Meier curves of LEPROT methylation level across cancer types. **(E)** The response rates to the PD-1 inhibitor in low and high groups stratified by median LEPROT expression in the GSE67501 data set (left panel) and the ROC for LEPROT expression to distinguish between responders versus nonresponders to the PD-1 inhibitor in GSE67501 with its AUC, which is 0.857 (right panel), “*” represents significance (*p* < .05) between two groups. **(F)** The response rates to the PD-1 inhibitor in low and high groups stratified by median LEPROT expression in the GSE79691 data set (left panel), and the ROC for LEPROT expression to distinguish between responder versus nonresponder to the PD-1 inhibitor in GSE79691 with its AUC of 0.857 (right panel), “ns” represents not significant (*p* > .05) between two groups.

Prognostic markers were biomarkers that were heterogeneously expressed in cancer samples with distinct patient outcomes. Identifying prognostic markers would help to group patients and guide precise drug discovery. Common prognostic markers were tumor suppressor, proto-oncogene, and TME components. Considering LEPROT was highly correlated with these common prognostic markers, we were curious if LEPROT could also provide prognostic value in patients. Thus, Cox analyses were performed for OS and DSS of LEPROT across pan-cancer types. The forest plots indicate that higher LEPROT was linked to worse OS and DSS in PAAD, CESC, LGG (*p* < .0001), and KICH (*p* < .05), worse OS in STAD (*p* < .05), worse DSS in BLCA (*p* < .05), and longer OS and DSS in SKCM (*p* < .05) and KIRC (*p* < .0001) (Figures 4A,B). Kaplan–Meier analysis showed an association of high LEPROT expression with better prognosis (both OS and PFS) in HNSC (*p* < .01), PAAD (*p* < .05), and STAD (*p* < .01), longer OS in KIRC (median OS HR = 0.54, p = 6e-5) and THCA (*p* < .05), and longer DFS in UCEC (*p* < .01) (Figures 4C,D). These indicate LEPROT might serve as potential prognostic markers for several cancer types.

### Predictive Values of the LEPROT Expression in Immune Checkpoint Inhibition Therapy

Considering that the LEPROT expression correlated with TII and immune checkpoints, we investigated whether it could imply patient responses to immune checkpoint inhibitors (CPIs). In KIRC (GSE67501), patients with higher LEPROT (above the median expression) had a significant (*p* = .022) lower response rate (0%, 0 in 5) to the PD-1 inhibitor nivolumab than those with low LEPROT (67%, 4 in 6) (Figure 4E, left panel). Although in SKCM (GSE79691), patients with higher LEPROT had a response rate of 80% (4 in 5) compared with a response rate of 40% (2 in 5) for patients with low LEPROT (Figure 4F, left panel), there was no significance between the two groups. The predictive role of LEPROT expression in CPI therapy was shown by ROC curves, and the area under the curve (AUC) was 0.875 in GSE67501 (Figure 4E, right panel) and 0.708 in GSE79691 (Figure 4F, right panel). These indicate that the LEPROT may influence CPI therapy response in different directions and has the potential to predict patient responses to CPI therapy.

## Discussion

In cancer cells, the molecular signaling processes were always aberrantly up/downregulated and/or functionally modified (Li et al., 2019) (Yang et al., 2020a; Barrett and Puré, 2020). As a result of conflicting reports on the impact of LEPROT on downstream signaling and cancer progression, and the lack of investigation on it, a bioinformatic analysis on LEPROT among multiple cancer types was, therefore, conducted for a broader understanding of the roles of LEPROT in cancers.

In line with previous reports (Rothzerg et al., 2020), LEPROT was widely expressed in human tissues and was downregulated in tumor tissues. Our study highlights the LEPROT expression as being significantly decreased in 12 cancer types, indicating that loss or downregulation of LEPROT expression could be associated with tumorigenesis in these cancers (Narrandes et al., 2018). As one of the effective mechanisms of gene modification, DNA methylation, whose abnormalities are related to abnormal expression of various genes, can activate or inhibit multiple signal transduction pathways, leading to oncogene or TSG alterations, thus inducing abnormal cell proliferation or apoptosis and promoting tumorigenesis (Huang et al., 2015) (Jin and Robertson, 2013). The negative correlations between the expression of LEPROT and its methylation indicate a regulation of LEPROT by DNA methylation in cancers. However, the DNMT family, which are currently thought to maintain (DNMT1) and induce DNA (DNMT3) or tRNA (DNMT2) methylation (Goll et al., 2006), were generally positively correlated with LEPROT expression. Thus, the function of DNMTs in this context cannot be simply concluded as catalyzing the transfer of methyl groups to DNA through their C-terminal catalytic domain and to subsequently suppress gene transcription (Espada et al., 2011; Jeltsch and Jurkowska, 2016). The regulation of LEPROT methylation levels might be performed by other factors, which are independent of the level of DNMTs. The reason for these rather contradictory results are still not entirely clear, but there are several possibilities: 1) DNMT1 and DNMT3a/b may also directly influence gene expression in a way that does not require the C-terminal catalytic domain (Espada et al., 2011) or by interacting with recruit histone deacetylases and histone methyltransferase (Milutinovic et al., 2004). 2) DNMT2, which is also known as transfer RNA (tRNA) aspartic acid methyltransferase 1 (TRDMT1) is suggested to have more activity on tRNA than DNA (Kaiser et al., 2017; Li et al., 2021), which may serve to stabilize specific tRNAs and conduct positive post-transcription regulation of aspartic acid-rich proteins instead of gene silencing. 3) DNMTs are suggested to be involved in many methylation-independent functions, for instance, the DNA damage repair (DDR) system, which are crucial for cancer cell survival. Both DNMT1 and DNMT2 were involved in DDRs by binding to DNA damage sites and re-repairing DDR molecules (Jin and Robertson, 2013). Therefore, the level of DNMTs is, to some extent, independent of the methylation level. 4) The DNA methylation pattern is suggested to be regulated by not only DNMTs, but also histone modifications (Moore et al., 2013; Hervouet et al., 2018), and translocation (Tet) enzymes (Kale et al., 2020). Thus, more factors should be taken into consideration when discussing the regulation of LEPROT methylation. However, due to the limitations of the study, the present study was only able to reflect a correlation rather than causation, and future causal studies are, therefore, required for full understanding of the relationships among LEPROT, DNA methylations, and DNMTs.

We noticed that the level of LEPROT was positively associated with both oncogenes and TSG. There are several possible explanations for these findings. First, the effects of oncogenes and TSGs are dynamic during tumor development. For instance, in tumor initiation, the activating mutation/overexpression of oncogenes or the inactivating mutation/decreased expression of TSGs yield tumorigenesis. During tumor progression, oncogenes confer to tumor growth and, at the same time, bring high oncogenic stress to tumor cells, characterized by replication overload and oxidative stress resulting in DNA damage, and ultimately lead to premature senescence. This process further activates the DDR system, which is exploited by tumor cells for escaping senescence. These DDR genes are majorly considered as TSG. Thus, during this period, LEPROT may be highly positively correlated with both oncogenes and some TSGs, especially those associated with DNA repair system. Second, it is shown that, regardless of whether a gene is recognized as an oncogene and TSG, its role and function depend not only on its own properties, but also on the cytogenetic background and signaling network under certain conditions (Stepanenko et al., 2013). Therefore, the correlation of LEPROT with oncogenes and TSGs may be related to the specific context and signaling networks, respectively.

Furthermore, the present study reveals that higher levels of LEPROT correlate with a worse prognosis in several cancers. It can be suggested due to the positive correlation between LEPROT and oncogenes, suggesting that high expression of LEPROT corresponds to a high replication rate in tumor cells, indicating a more aggressive cancer type and a worse prognosis in the patient. Meanwhile, oncogenes might induce cellular senescence (OIS) accompanied with senescence-associated secretory phenotype (SASP), which confers tumor-associated macrophages and CAFs to constitute an immunosuppressive microenvironment that allows the progression of the cancer (Toste et al., 2016; Liu et al., 2018; Yasuda et al., 2021). Moreover, cytokines secreted by senescent cells such as interleukin-6 (IL-6) and C-X-C pattern chemokine ligand 1 (CXCL1) are also shown to promote tumor development by stimulating endothelial cell proliferation, promoting angiogenesis, facilitating tumor cell invasion, or inducing cancer stem cell formation (Liu et al., 2018). However, overexpression of LEPROT is also shown to be positive correlated with patient survival and indicates favorable responses to immunosuppressive therapy in some tumors, such as SKCM. A possible explanation for that may be the massive recruitment of immune cells by SASP, which constitutes a “hot” TME, acts to eliminate tumor cells, and also allows patients to respond well to immunosuppressive agents (Liu et al., 2018; Kale et al., 2020).

The study reveals for the first time that there are intensive correlations of LEPROT expression with infiltrating immune cells and CAFs, which are the critical components in the TME (Quail and Joyce, 2013; Kato et al., 2018; Chen and Song, 2019). Infiltrating immune cells and CAFs interact with each other, and both could communicate with cancer cells (Kato et al., 2018; An et al., 2020). As determining components of conducting tumor immune response, infiltrating immune cells were remarkably correlated with LEPROT expression in tumor tissues, suggesting the similar features of LEPROT in tumor immune response. However, the effects of LEPROT on the components of TII were not always consistent; they were diverse regarding different types of immune cells or different algorithms. Notably, the positive correlation of LEPROT expression was robust and consistent with CAF levels. Recall that CAFs were the most abundant stromal cells in the TME and could facilitate tumor initiation, progression, and angiogenesis by supporting tumor cell growth and extracellular matrix remodeling and by mediating tumor-promoting inflammation genes (Kato et al., 2018; Chen and Song, 2019). In the TME, CAFs remodeled the extracellular matrix and increase the tissue stiffness, which favored cancer progression and resulted in unfavorable patient outcomes (Chen and Song, 2019). CAFs also secreted various inflammatory cytokines and growth factors, including interleukin-6 (IL-6), chemokine (C-X-C motif) ligands (CXCLs), and transforming growth factor beta (TGF-β), to activate oncogenic pathways (Inoue et al., 2019). Complex influences of CAFs on tumor immunity and immunotherapy responses have been revealed (Wong et al., 2019). For instance, CAFs might increase the expression of PD-L1 in carcinoma cells and recruit immune cells, mainly immunosuppressive cells, into the TME (Chen and Song, 2019). As one of the ICMs, PD-L1 can be induced by cytokines in the TME with inflammation signaling (Chen et al., 2019b). ICMs are suggested to profoundly interact with TME and influence the effect of immune therapy (Wang et al., 2019a; Wang et al., 2019b; Zhang et al., 2020b), playing key roles in immune checkpoint inhibition. In the current study, a positive correlation between LEPROT expression and PD-L1 was observed across all cancer types and indicates a role of LEPROT in tumor escaping in cancers. In addition, TLRs were shown to be intensively correlated with LEPROT in the study and might be another factor contributing to the complex roles of LEPROT. TLRs are shown to stimulate the adaptive immune system and increase antitumor antigen-specific T cells in cancer via upregulating co-stimulatory signals (Adams, 2009; Salem, 2011). Various TLR ligands are also reported to increase the proliferation of cancer cells via cytokines such as IL-6 and subsequent signaling (Adams, 2009). Therefore, higher TLRs, generally accompanied with higher LEPROT, might not only facilitate antitumor immunity, but also promote cancer cell proliferation. To sum up, we found LEPROT had remarkably constant correlations with the TME components, including immune regulating molecules, tumor immune cell infiltration, and CAFs in a context-independent way. Nevertheless, the overall roles of LEPROT in TME and tumor immunity are multidimensional.

The predictive value of the LEPROT expression in immune checkpoint inhibition therapy was variate. In KIRC, the expression of LEPROT was significantly and positively correlated with PD-L1 and compromised the patients’ responses to the PD-1 inhibitor. Inconsistent with this, high expression of LEPROT was beneficial for OS and DFS in KIRC patients. These suggest that patients with low expression of LEPROT in KIRC should be considered first for immunotherapy. However, in SKCM, the expression of LEPROT also had a strong correlation with PD-L1, but appeared to favor the effect of the PD-1 inhibitor. The inconsistency among cancer types suggests a contradictory and context-dependent role of LEPROT in predicting the immunotherapy response and the overall outcomes. Further investigations are required to reveal its clinical value, including as an indicator of responses to tumor immunotherapies in a cancer-specific way given the multidimensional roles of LEPROT and the complex nature of cancers.

It is reported that dysregulation of LEPROT is associated with various bone inflammation diseases through key cytokines, such as tumor necrosis factor alpha (TNF-α) and interleukin 6 (IL-6) (Rothzerg et al., 2020). It is suggested to be implicated in osteosarcoma initiation and metastasis through upregulating the level of IL-6 and TNF-α. Generally, IL-6 signals through a cell-surface type I cytokine receptor complex consisting of the ligand-binding IL-6Rα chain and the signal-transducing component gp130, which is also known as IL-6 signal transducer (IL6ST) (Rothzerg et al., 2020). Consistent with previous studies (Sanz-Moreno et al., 2011; Looyenga et al., 2012; Pilati and Zucman-Rossi, 2015), the IL6ST gene, which was the top co-regulated gene, conducted its function via activation of the JAK1/2/STAT3 pathway in our study, and the mRNA levels of IL6ST companioned with its targets JAK1/2 and STAT3 were positively related with LEPROT mRNA levels in not only SARC but also in almost all pan-cancer studies. As a matter of fact, JAK-STAT signaling molecules and pathways were also suggested by PPI analysis. Therefore, we highlighted the IL6ST/JAK/STAT pathway in the effect of LEPROT in cancer, which was profoundly involved in the proliferation of cancer cells, immune regulation, and CAF activation and function (Hirano et al., 2000; Carbia-Nagashima and Arzt, 2004; Yu et al., 2009; Calon et al., 2012; Amin et al., 2017; Jones and Jenkins, 2018; Biffi et al., 2019; Yang et al., 2020b; Cheteh et al., 2020).

Signal transduction in cancer cells is profoundly different from that in normal cells, and the effects of specific molecules or pathways on cell processes may exert different cellular responses in tumors than in normal tissue (Sanz-Moreno et al., 2011; Pascale et al., 2020). Differences in signal pathways are commonly observed in distinct cancer types as well (Cui et al., 2020; Cui et al., 2021), and thus, the cancer-related functions or interactions of proteins of interest are usually investigated in a cancer-specific way. In the present study, we found that there was indeed heterogeneity in the expression level, the correlation with oncogenes or tumor suppressor genes of LEPROT across cancer types, but at the same time, we were surprised to notice the remarkably consistent correlation of LEPROT and effector T cells, resident memory T cells, Th1 cells, and CAFs across all cancer types. Moreover, regardless of the regulation of LEPROT in cancer, it maintained a high correlation with the IL6ST/JAK1/2/STAT3 pathway. These findings revealed that LEPROT has robust relationships with inflammatory genes and TME elements, indicating a role of LEPROT in regulating inflammatory or immune signals.

## Data Availability

The original contributions presented in the study are included in the article/Supplementary Material, further inquiries can be directed to the corresponding author.
