# Supplementary figures and images for: Leptin Receptor Overlapping Transcript (LEPROT) Is Associated with the Tumor Microenvironment and a Prognostic Predictor in Pan-Cancer
Source: Front Genet. 2021 Nov 11;12:749435. doi: 10.3389/fgene.2021.749435 (PMC8596502; doi:10.3389/fgene.2021.749435)

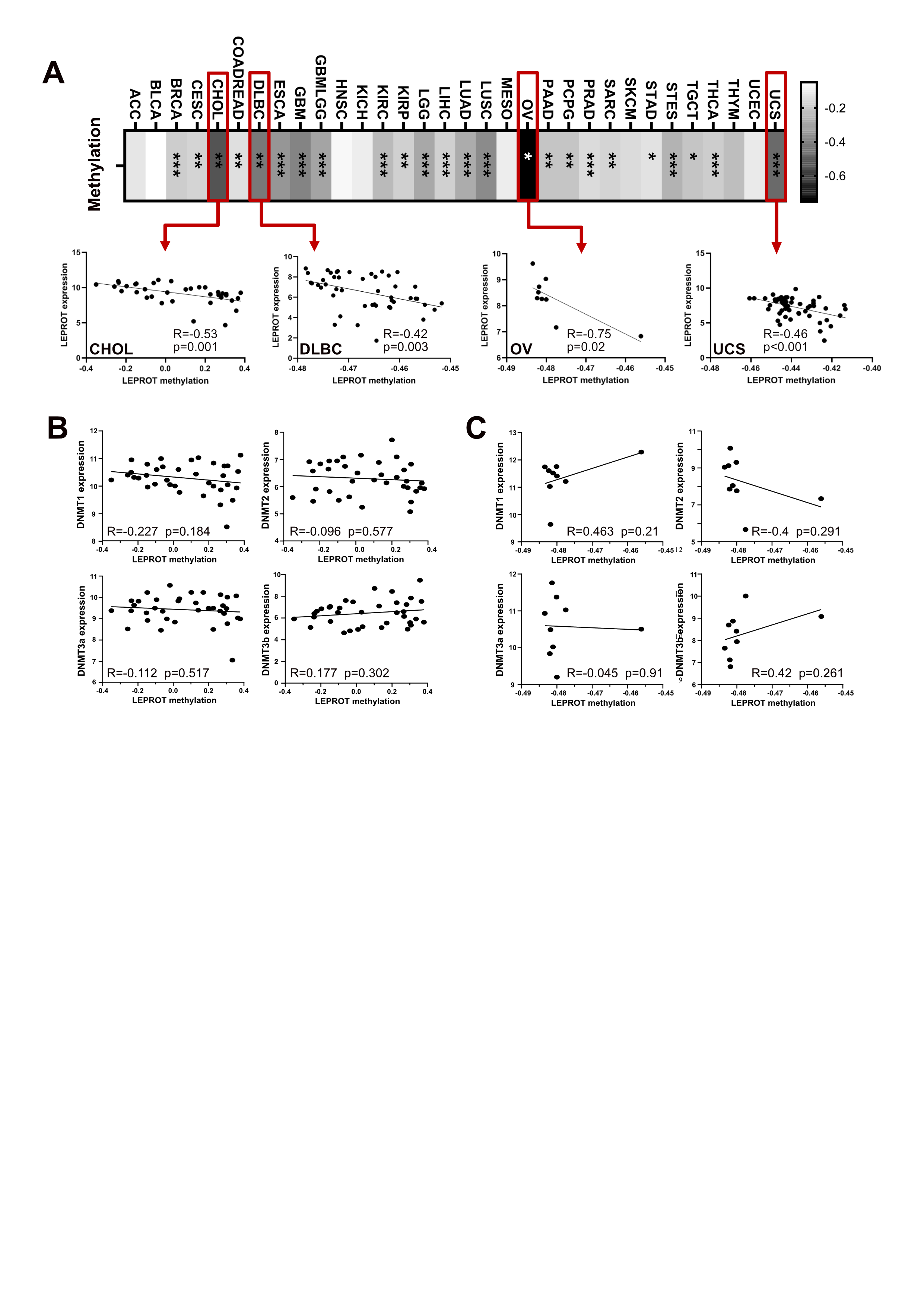

Supplement: Supplementary file 1 [file Image3.TIFF]

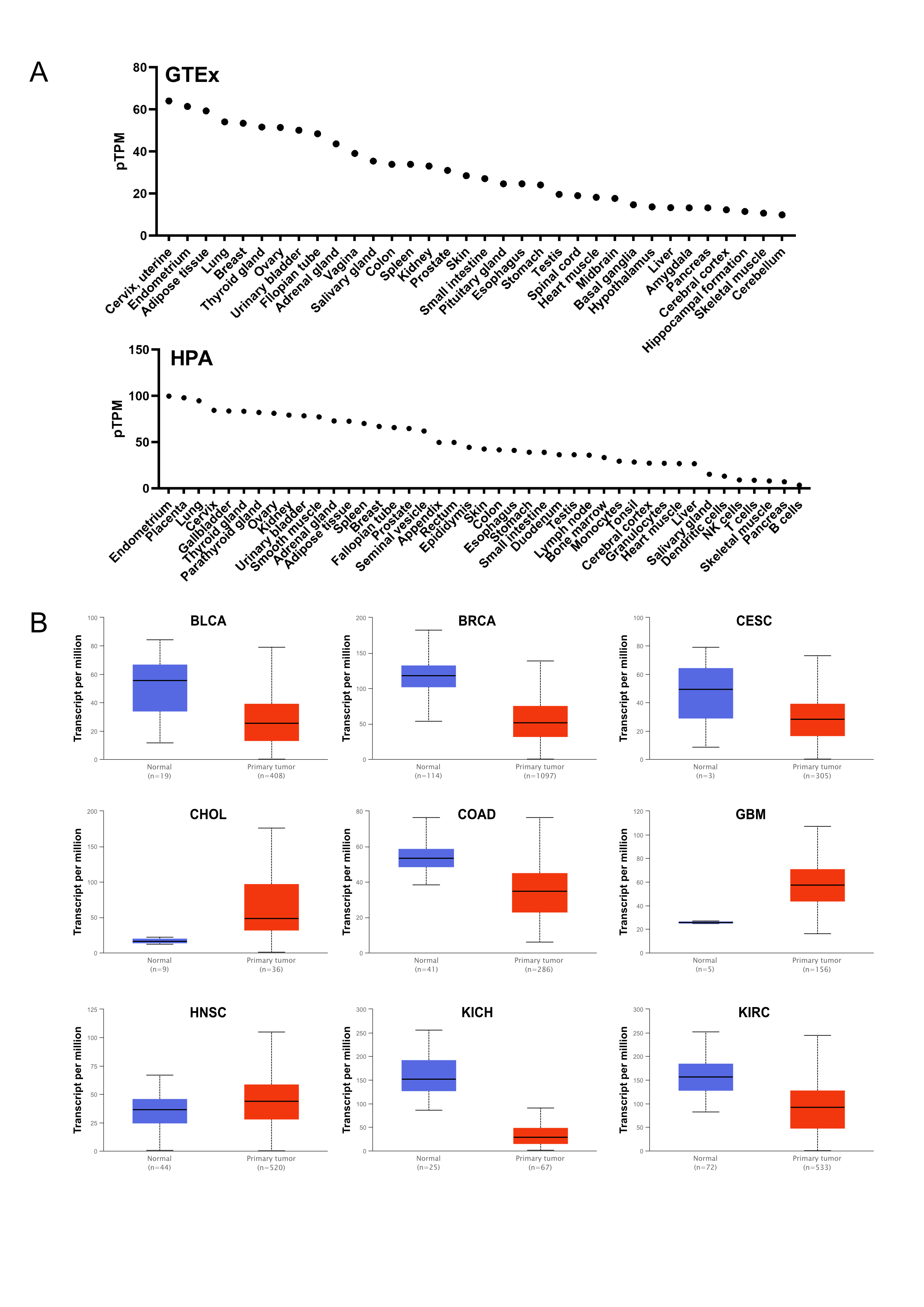

Supplement: Supplementary file 2 [file Image1.TIFF]

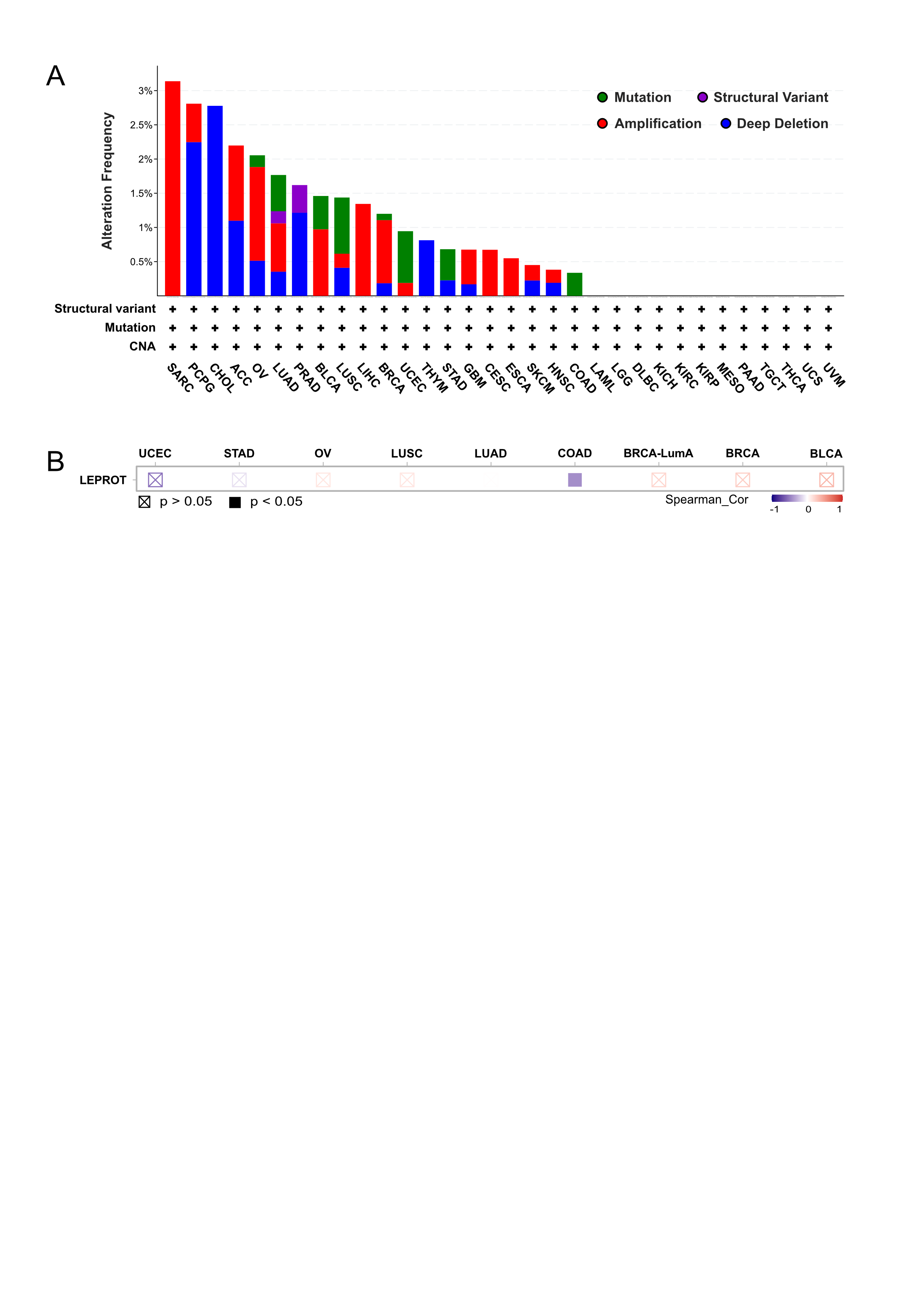

Supplement: Supplementary file 3 [file Image2.TIFF]

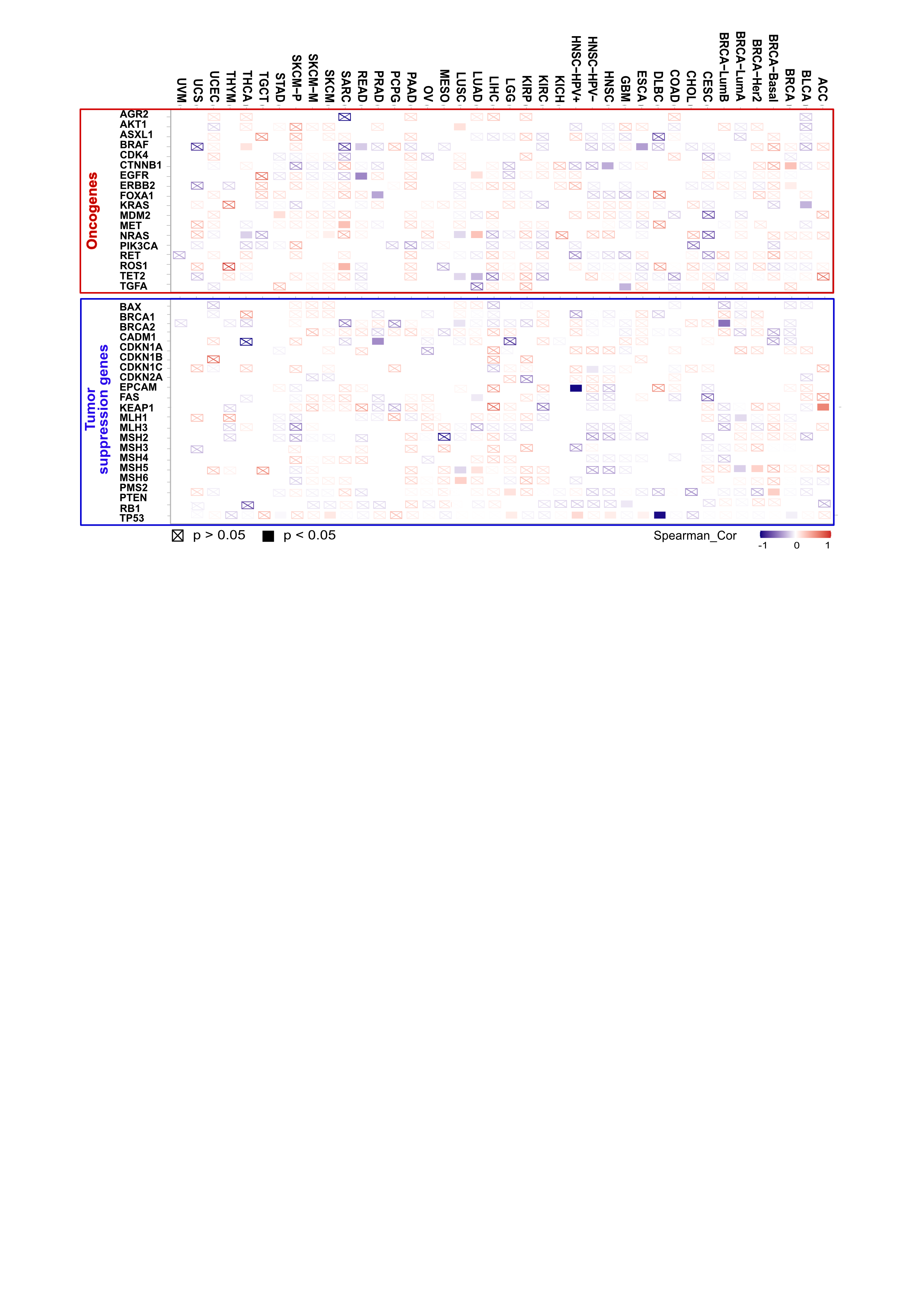

Supplement: Supplementary file 4 [file Image4.TIFF]
